# Post-transplant absolute lymphocyte count predicts early cytomegalovirus infection after heart transplantation

**DOI:** 10.1038/s41598-020-80790-4

**Published:** 2021-01-14

**Authors:** Minjae Yoon, Jaewon Oh, Kyeong-Hyeon Chun, Chan Joo Lee, Seok-Min Kang

**Affiliations:** Cardiology Division, Severance Cardiovascular Hospital, Cardiovascular Research Institute, Yonsei University College of Medicine, Seoul, South Korea

**Keywords:** Cardiovascular biology, Heart failure

## Abstract

Immunosuppressive therapy can decrease rejection episodes and increase the risk of severe and fatal infections in heart transplantation (HT) recipients. Immunosuppressive therapy can also decrease the absolute lymphocyte count (ALC), but the relationship between early post-transplant ALC and early cytomegalovirus (CMV) infection is largely unknown, especially in HT. We retrospectively analyzed 58 HT recipients who tested positive for CMV IgG antibody and received basiliximab induction therapy. We collected preoperative and 2-month postoperative data on ALC and CMV load. The CMV load > 1200 IU/mL was used as the cutoff value to define early CMV infection. Post-transplant lymphopenia was defined as an ALC of < 500 cells/μL at postoperative day (POD) #7. On POD #7, 29 (50.0%) patients had post-transplant lymphopenia and 29 (50.0%) patients did not. The incidence of CMV infection within 1 or 2 months of HT was higher in the post-transplant lymphopenia group than in the non-lymphopenia group (82.8% vs. 48.3%, *P* = 0.013; 89.7% vs. 65.5%, *P* = 0.028, respectively). ALC < 500 cells/μL on POD #7 was an independent risk factor for early CMV infection within 1 month of HT (odds ratio, 4.14; 95% confidence interval, 1.16–14.77; *P* = 0.029). A low ALC after HT was associated with a high risk of early CMV infection. Post-transplant ALC monitoring is simple and inexpensive and can help identify patients at high risk of early CMV infection.

## Introduction

Opportunistic infections represent a significant problem after transplantation. Cytomegalovirus (CMV) infection remains the most common infections after solid organ transplantation, resulting in significant morbidity and related mortality^1–3^. Current antiviral agents and preventive strategies after solid organ transplantation have led to a decrease in the incidence of CMV diseases, and current guidelines recommend CMV prophylaxis or preemptive therapy after transplantation^4, 5^.

In heart transplantation (HT), the appropriate use of immunosuppressive agents is crucial to prevent graft rejection^6^. Induction therapy, an intense prophylactic treatment strategy, is thought to be related to lower acute rejection in the early post-transplant period. Basiliximab, a monoclonal antibody for interleukin-2 (IL-2), and anti-thymocyte globulin (ATG), a polyclonal anti-lymphocyte antibody, are the most commonly used induction drugs for HT^7, 8^. These immunosuppressive agents can decrease rejection episodes^9^, but they can increase the risk of severe and fatal infections in HT recipients^8, 10–12^. In South Korea, there is a high prevalence (> 90%) of CMV seropositivity among the general population, similar to other countries^13, 14^. Therefore, immunosuppressive therapy including induction therapy can be related to a higher risk of CMV infection after HT^15, 16^.

Immunosuppressive therapy after transplantation can decrease the absolute lymphocyte count (ALC) and their functions^17^. A low CD4 + cell count represents a major risk factor for the development of opportunistic infections in HIV patients^18^. Hence, it is a standard of care to use the CD4 + cell count clinically to guide the initiation of prophylactic HIV antimicrobial agents. The lymphocyte count is a simple and inexpensive measurable value. The general concept that low lymphocyte count indicates inadequate reserves to mount an appropriate immunologic response can be extended to opportunistic infections after transplantation. Several studies have considered the relevance of lymphocyte count for identifying and monitoring patients at risk for post-transplant-related infections^19–23^. Recent studies have suggested that pre-transplant low ALC could be a risk factor for CMV infections after transplantation^24, 25^. However, the relationship between early post-transplant ALC and early CMV infections is largely unknown, especially in HT.

We hypothesized that a low ALC after HT correlates with a higher risk of early CMV infection. Therefore, we investigated the peri-transplant ALC and its association with the incidence of early CMV infection in HT recipients.

## Methods

### Study design

We performed a retrospective analysis of patients who underwent HT from January 2013 to August 2016 at a tertiary university-affiliated hospital (Severance cardiovascular hospital, Seoul, South Korea). During this period, all recipients were CMV immunoglobulin G (IgG) antibody positive (R +), and they all received basiliximab induction therapy, followed by triple maintenance immunosuppressive therapy (tacrolimus, mycophenolate mofetil, and corticosteroids). Patients received basiliximab induction (20 mg intravenous [IV] infusion) on postoperative day (POD) #0 and #4. Immediately after the operation, 500 mg of methylprednisolone was administered, followed by 125 mg every 8 or 12 h for 3 days with the administration of tacrolimus and mycophenolate mofetil. Then, 1 mg/kg/day of prednisolone was administered and tapered to 5 mg/day weekly. From a total of 70 patients, we excluded the patients who died before POD #14, had no CMV load data, or were aged < 16 years (Supplementary Figure S1 Online). Finally, we enrolled 58 patients who underwent HT. All patients were followed up till 60 days after HT.

In our institute, for CMV prevention, we used preemptive therapy in CMV seropositive HT recipients (R +) according to the current guidelines^5, 26^. Preemptive therapy was defined as the administration of anti-CMV agents only to asymptomatic patients with evidence of early CMV replication to prevent CMV disease^27^. For CMV preemptive therapy, we used intravenous ganciclovir (10 mg/kg/day) or oral valganciclovir (1800 mg/day), with the doses adjusted for renal impairment until the resolution of CMV DNAemia, with a minimum of 2 weeks of treatment.

Baseline demographic data and comorbidities were evaluated. In addition, CMV load, anti-CMV agent use, peri-transplant ALC, and acute graft rejection within 2 months after HT were collected for analysis. Endomyocardial biopsy was performed to confirm the diagnosis of acute graft rejection according to the International Society for Heart and Lung Transplantation (ISHLT) grading system, similarly as our previous study—Grade 0, no rejection; Grade 1R, mild; Grade 2R, moderate; or Grade 3R, severe^28, 29^. This study was approved by the Institutional Review Board of Yonsei University Hospital (4–2013-0665) and was conducted in accordance with the Declaration of Helsinki. Informed consent from the patients was obtained.

### Definition of CMV infection and the measurement of CMV load

In accordance with the guidelines^5, 30^, we defined CMV infection as evidence of CMV replication regardless of symptoms, defined as virus isolation or detection of viral proteins or nucleic acid in any body fluid or tissue specimen. CMV disease was defined as evidence of CMV infection with attributable symptoms.

We performed quantitative nucleic acid testing by extracting CMV DNA from whole blood. Real-time polymerase chain reaction (PCR) for CMV DNA was performed using a Light Cycler 480 (Roche Diagnostics, Germany) and Bio-Core CMV Quantification real-time PCR kit (Bio-Core, Korea)^31^. For the standardization of results, the World Health Organization International Standard for human CMV for nucleic acid amplification techniques was used. The CMV load data was reported in IU/mL and measured at least weekly until 2 months after HT.

Recently, the guidelines reported the need for establishing a universal viral load threshold for initiating preemptive therapy^5, 32^. However, the standardization of specific cutoff values is limited because of variations in the performance of tests and the diversity of the patient population. By considering previous studies about the viral load threshold^33–36^, our institute has used a CMV load > 1200 IU/mL a**s** a threshold for initiating preemptive therapy. Although any detectable CMV load (e.g., 1000 IU/mL) is included in the definition of CMV infection according to the guidelines, we use the “early CMV infection” term in this study when the highest value of the CMV load within 1 or 2 months after HT is more than 1200 IU/mL.

### Measurement of ALC

We measured ALC by collecting blood samples. ALC was calculated using the following formula: ALC = White blood cell (WBC) count × lymphocyte percent. We collected preoperative and 2-month postoperative ALC data. Post-transplant lymphopenia was defined as an ALC of < 500 cells/μL based on previous studies^24^. In sensitivity analysis, we performed similar analysis by changing the cutoff value of ALC from 500 to 610 cells/μL based on another recent study^37^. We stratified patients by the presence of post-transplant lymphopenia on POD #7.

### Statistical analysis

Descriptive statistics were used to characterize baseline characteristics and comorbidities. Categorical variables are reported as frequencies (percentages). Continuous variables are expressed as medians with interquartile range. The categorical variables were compared using Fisher’s exact test or the Pearson chi-square test, and continuous variables were compared using the Mann–Whitney U-test. Risk factors for early CMV infection were identified using univariable and multivariable binary logistic regression analyses. All tests were two-tailed, and differences were considered significant at *P* < 0.05. Statistical analyses were performed using SPSS version 25.0 statistical package (SPSS Inc., Chicago, IL, USA).

## Results

### Baseline characteristics of heart transplantation

The baseline characteristics of patients are listed in Table 1. There were 29 (50%) patients with post-transplant lymphopenia on POD #7, and 29 (50%) patients without post-transplant lymphopenia on POD #7. There were no significant differences in terms of the various parameters of donors. The recipient age of the post-transplant lymphopenia group was significantly greater than that of the non-lymphopenia group (52 [44–57] years vs 46 [33–53] years, *P* = 0.048). There was no significant difference between the groups in other baseline co-morbidities of recipients. Pre-transplant ALC and ALC on POD #7 were significantly lower in the post-transplant lymphopenia group than in the non-lymphopenia group (1110 [630–1330] cells/μL vs. 1480 [1030–1910] cells/μL, *P* = 0.003, for pre-transplant ALC and 330 [250–380] vs. 840 [690–1100] cells/μL, *P* < 0.001, for ALC on POD #7, respectively). There was no significant difference in the underlying heart diseases of recipients between the groups.

**Table 1:** Baseline characteristics of the study population stratified by the ALC on POD #7.

Variable	All patients (n = 58)	ALC < 500 (n = 29)	ALC ≥ 500 (n = 29)	*P* value
**Donors**				
Age (years)	41 (30–46)	41 (34–46)	38 (28–45)	0.141
Female sex, n (%)	24 (41)	10 (35)	14 (48)	0.286
Height (m)	1.67 (1.61–1.75)	1.67 (1.63–1.75)	1.68 (1.60–1.75)	0.601
Weight (kg)	66.0 (58.1–75.8)	69.0 (58.1–76.4)	65.0 (58.7–70.3)	0.750
BMI (kg/m^2^)	23.5 (21.5–25.3)	23.6 (21.6–25.6)	23.4 (21.6–25.1)	0.926
BSA (m^2^)	1.77 (1.63–1.91)	1.80 (1.59–1.94)	1.73 (1.63–1.84)	0.686
LVEF (%, median [IQR])	60 (56–65)	58 (55–65)	61 (58–70)	0.068
**Recipients**				
Age (years)	50 (38–57)	52 (44–57)	46 (33–53)	0.048
Female sex, n (%)	38 (66)	21 (72)	17 (59)	0.269
Height (m)	1.66 (1.59–1.71)	1.67 (1.58–1.70)	1.64 (1.60–1.71)	0.963
Weight (kg)	60.0 (53.7–69.6)	60.5 (55.5–70.0)	60.0 (52.0–69.1)	0.479
BMI (kg/m^2^)	22.8 (20.1–24.9)	23.3 (20.2–25.2)	21.2 (20.5–24.0)	0.371
BSA (m^2^)	1.67 (1.54–1.82)	1.68 (1.59–1.84)	1.65 (1.53–1.82)	0.549
Pre-transplant LVEF (%, median [IQR])	19 (16–27)	19 (16–25)	21 (16–27)	0.870
Previous heart surgery, n (%)	15 (26)	9 (31)	6 (21)	0.368
Hypertension, n (%)	19 (33)	11 (38)	8 (28)	0.401
Diabetes mellitus, n (%)	13 (22)	6 (21)	7 (24)	0.753
CMV-seropositive, n (%)	58 (100)	29 (100)	29 (100)	–
Pre-transplant total WBC count (cells/μL, median [IQR])	7160 (3750–10,510)	6380 (3510–10,460)	7390 (5310–10,380)	0.451
Pre-transplant ALC (cells/μL, median [IQR])	1250 (800–1710)	1110 (630–1330)	1480 (1030–1910)	0.003
ALC on postoperative day 7, (cells/μL, median [IQR])	470 (320–840)	330 (250–380)	840 (690–1100)	< 0.001
Underlying heart disease, n (%)				
Ischemic cardiomyopathy	13 (22)	6 (21)	7 (24)	0.999
Dilated cardiomyopathy	19 (33)	9 (31)	10 (35)	0.999
Valvular heart disease	6 (10)	4 (14)	2 (7)	0.666
Congenital heart disease	5 (9)	2 (7)	3 (10)	0.999
Others	15 (26)	8 (28)	7 (24)	0.881

Values are expressed in numbers (%), or median (interquartile range). ALC = absolute lymphocyte count, BMI = body mass index, BSA = body surface area, CMV = cytomegalovirus, POD = postoperative day, WBC = white blood cell.

### Time course of ALC following HT

**Figure 1:**
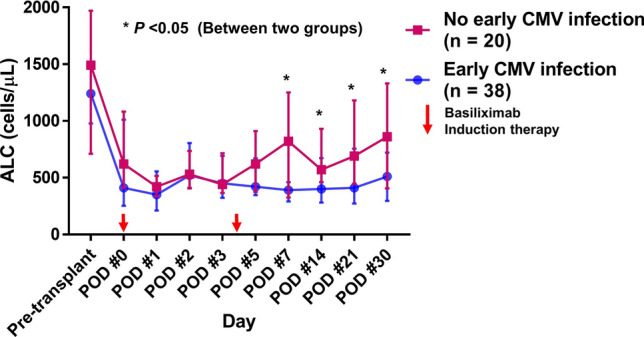
Time course of median value of ALC following heart transplantation. ALC = absolute lymphocyte count, CMV = cytomegalovirus, POD = postoperative day.

**Figure 2:**
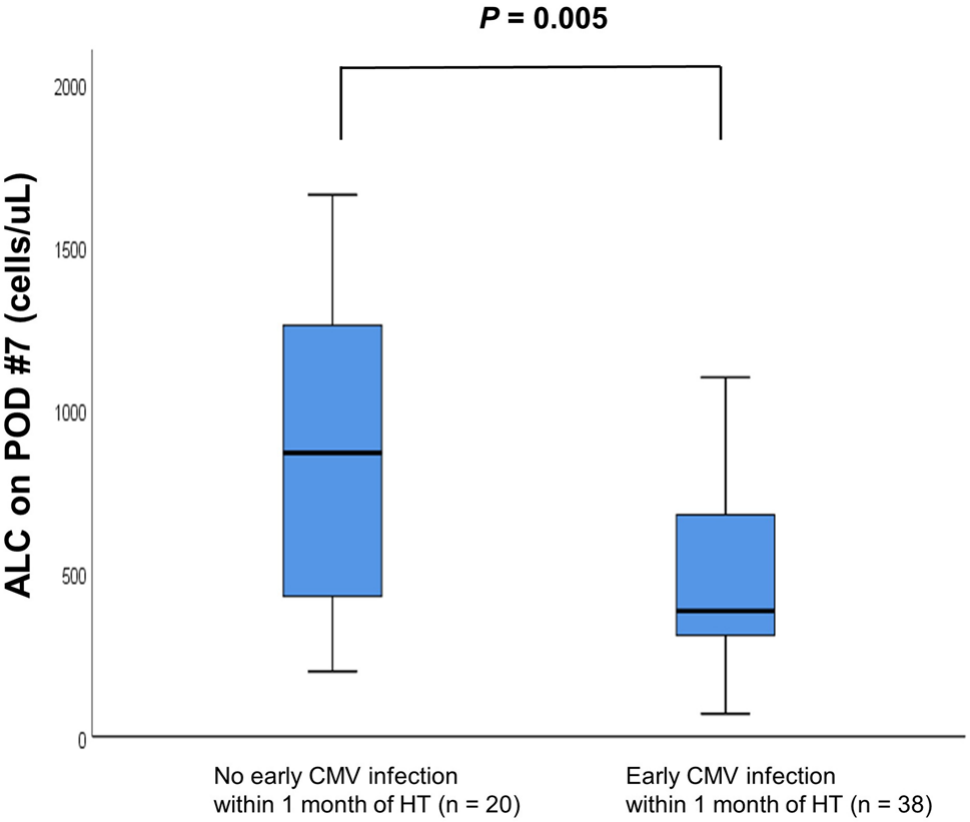
Distribution of ALC on postoperative day 7 in patients who had early CMV infection within one month after HT (n = 38), and those who did not (n = 20). ALC = absolute lymphocyte count, CMV = cytomegalovirus, HT = heart transplantation.

The time courses of median value of ALC following HT are shown in Fig. 1. Patients were stratified by the occurrence of early CMV infection within 1 month of HT. The pre-transplant ALC levels were not significantly different between the groups. In addition, there were no significant differences in ALC on PODs #0, 1, 3, and 5. However, ALC on PODs #7, 14, 21, and 30 were significant lower in the early CMV infection group than in the no early CMV infection group. The distribution of ALC on POD #7 in patients who had early CMV infection within 1 month after HT (n = 38) and those who did not (n = 20) is shown in Fig. 2. The median ALC in the early CMV infection group was significantly lower than that in the no early CMV infection group (380 [290–680] cells/μL vs. 840 [340–1240] cells/μL, *P* = 0.005).

### Post-transplant CMV infection outcomes

Table 2 summarizes the post-transplant outcomes stratified by ALC on POD 7. The post-transplant lymphopenia group (ALC on POD #7 < 500 cells/μL) had a higher incidence of CMV infection within 1 or 2 months of HT than the non-lymphopenia group (1 month: 83% vs. 49%, *P* = 0.013; 2 months: 90% vs. 66%, *P* = 0.028, respectively). ALC on PODs #14, 21, and 30 were significantly lower in the post-transplant lymphopenia group than in the non-lymphopenia group (all *P* < 0.05). In addition, the prevalence of allograft rejection within 2 months of HT was not significantly different between groups.

**Table 2:** Post-transplant outcomes stratified by the ALC on POD #7.

	ALC < 500 (n = 29)	ALC ≥ 500 (n = 29)	*P* value
Early CMV infection within 1 month of HT, n (%)	24 (83)	14 (49)	0.013
Early CMV infection within 2 months of HT, n (%)	26 (90)	19 (66)	0.028
CMV disease within 2 months of HT, n (%)	4 (14)	1 (3)	0.349
Anti-CMV therapy initiated within 2 months of HT, n (%)	26 (90)	21 (72)	0.180
Valganciclovir, n (%)	1 (3)	2 (7)	0.999
Ganciclovir, n (%)	25 (86)	19 (66)	0.028
Time from transplant to CMV onset (days, median [IQR])	18 (11–24)	22 (15–36)	0.019
Acute graft rejection within 2 months of HT by endomyocardial biopsy, n (%)			
Grade 0	12 (52)	10 (40)	0.080
Grade 1R,	10 (44)	15 (60)	0.123
Grade 2R	1 (4)	0 (0)	0.490
Grade 3R	0 (0)	0 (0)	–
ALC on POD #14, (cells/μL, median [IQR])	380 (240–540)	690 (430–920)	0.001
ALC on POD #21, (cells/μL, median [IQR])	340 (240–550)	730 (510–1040)	< 0.001
ALC on POD #30, (cells/μL, median [IQR])	390 (240–540)	850 (640–1260)	< 0.001

Values are expressed in numbers (%) or the median (interquartile range). ALC = absolute lymphocyte count, CMV = cytomegalovirus, HT = heart transplantation, POD = postoperative day.

### Post-transplant low ALC as a risk factor for early CMV infection

The logistic regression of risk factors for early CMV infection within 1 month of HT is shown in Table 3. Univariable regression analysis revealed a significant association between early CMV infection within 1 month of HT and the following variables: age, decrease in ALC on POD #7, an ALC of < 500 cells/μL on POD #7, and an ALC of < 610 cells/μL on POD #7. Multivariable regression analysis was performed by using total three variables: age, sex, and ALC < 500 (or 600) cells/μL on POD #7. Multivariable analysis revealed that an ALC of < 500 cells/μL on POD #7 was an independent risk factor for the early CMV infection within 1 month of HT after adjusting age and sex (Model 1: odds ratio [OR], 4.14; 95% confidence interval [CI], 1.16–14.77; *P* = 0.029). In sensitivity analysis, an ALC cutoff value of < 610 cells/μL was used, and among the 3 parameters retained in a multivariable regression analysis model 2, age and ALC < 610 cells/μL on POD #7 prevailed as independent predictors for the early CMV infection within 1 month of HT. Similar logistic regression analysis was performed on the risk factors for early CMV infection within 2 months of HT (Supplementary Table S1 online). In Univariable regression, an ALC of < 500 cells/μL on POD #7 was associated with higher early CMV infection within 2 months of HT (OR, 4.56; 95% CI, 1.10–18.86; *P* = 0.036). Total leukocyte count on pre-transplant or POD #7 were not the predictor of early CMV infection within 1 month in our study (Supplementary Table S2 online).

**Table 3:** Univariable and multivariable logistic regression analysis of risk factors for early CMV infection within 1 month of heart transplantation.

	Univariable analysis			Multivariable analysis (Model 1)			Multivariable analysis (Model 2)		
	OR	95% CI	*P* value	OR	95% CI	*P* value	OR	95% CI	***P*** value
Age	1.06	1.01–1.10	0.011	1.05	1.01–1.11	0.037	1.06	1.00–1.11	0.042
Female	0.80	0.27–2.38	0.685	1.62	0.42–6.22	0.484	1.48	0.39–5.59	0.563
Decrease in pre-transplant ALC (per 100 cells/μL increment)	1.08	0.99–1.18	0.095						
Decrease in ALC on POD #7 (per 100 cells/μL increment)	1.22	1.05–1.41	0.009						
ALC < 500 on POD #7 versus ALC ≥ 500 cells/μL	5.14	1.53–17.20	0.008	4.14	1.16–14.77	0.029			
ALC < 610 on POD #7 versus ALC ≥ 610 cells/μL	5.20	1.62–16.78	0.006				4.01	1.12–14.08	0.025

ALC = absolute lymphocyte count, CI = confidence interval, CMV = cytomegalovirus, OR = odds ratio, POD = postoperative day.

## Discussion

In this study, we performed analysis with the purpose of obtaining information regarding the ALC after HT and its relationship with the incidence of early CMV infection. Our principal findings are as follows: (1) ALC on PODs #7, 14, 21, and 30 was significantly lower in the early CMV infection group than that in the no early CMV infection group; (2) post-transplant lymphopenia group on POD #7 had a higher incidence of early CMV infection than the non-lymphopenia group; and (3) an ALC of < 500 cells/μL on POD #7 was an independent risk factor for early CMV infection in HT. These results can be translated clinically to indicate that close monitoring of ALC after HT may help identify patients at high risk of early CMV infection.

A recently published study showed that a low ALC at the end of CMV treatment completion was an independent predictor for recurrent CMV diseases in solid organ transplant recipients^23^, compatible with our findings. Another study from the Mayo clinic also reported that nonspecific and CMV-specific CD8 + T cell functions correlated with the course of CMV after solid organ transplant^38^. These findings may be biologically plausible considering the importance of T cell immunity in maintaining CMV latency in transplant recipients. Post-transplant ALC monitoring is simple and inexpensive; hence, it could help identify patients at high risk of early CMV infection, considering the high cost of CMV load monitoring.

In general, ALC is affected not only by induction therapy but also by anti-CMV agents, other medications used for HT (e.g., mycophenolate mofetil, steroid), or CMV infection itself. Considering most preemptive therapies for CMV are initiated at least 2 weeks after HT in our study, we focused on the ALC on POD #7. Thus, the effect of anti-CMV agents or CMV infection on ALC on POD #7 could be minimalized. Further, ALC on POD #7 is ideal for reflecting the effect of induction therapy because patients received basiliximab induction therapy on PODs #0 and #4. The consequence of our study is that low ALC after heart HT can be used in predicting early CMV infection. Therefore, more frequent monitoring of the CMV load or early aggressive initiation of anti-CMV treatments for preemptive therapy could be considered in post-transplant lymphopenia group.

Some studies on induction therapy after transplantation may suggest that we need more individualized induction strategies for decreasing the risk of both infection and rejection. For instances, considering the long half-life of basiliximab (14–21 days), single (vs. double dose) or decreased (two 10 mg vs. 20 mg) doses of basiliximab induction therapy could be individualized options for selected HT recipients^39, 40^. In addition, some institutions have used the ALC-guided ATG dosing strategy (e.g., targeted to < 100–200 cells/mm^3^) after ATG induction in solid organ transplantation (especially the kidney). It has also been mentioned in the current ISHLT guidelines for the care of HT recipients as class IIb indications and the level of evidence C^6^. Therefore, serial monitoring of ALC during the peri-operative period may be another individualized option for immunologic monitoring in HT recipients to decrease the risk of infection and rejection.

CMV infection after transplantation is associated with numerous indirect effects, such as an increased risk of acute rejection, chronic allograft failure, and other opportunistic infections^41–43^. Recently, some studies have reported a significant association between CMV infection and cardiac allograft vasculopathy (CAV); however, in one study, a significant effect of CMV infection on the risk of CAV was seen only among HT recipients who had CMV breakthrough infection while receiving prophylaxis^44–46^. Because of its negative impact, CMV prevention is a major focus of post-transplant management, which can be accomplished either by preemptive or antiviral prophylaxis. Hence, serial monitoring of ALC can decrease severe CMV disease and result in a low incidence of allograft rejection and CAV after transplantation. Further studies are warranted to prove this hypothesis.

### Limitations

This study has some limitations. First, this study was a single-center, retrospective study with statistical power limited by the number of cases and outcomes. Second, we did not have any data for HT patients with ATG induction therapy. Third, the donor CMV serostatus could not be investigated. However, considering that the current guidelines recommend CMV prophylaxis or preemptive therapy for seropositive recipients regardless of donor serostatus, it was reasonable to use preemptive therapy in all CMV seropositive recipients in our study. Fourth, our institution used a comparably low CMV load (= 1200 IU/mL) as threshold for pre-emptive therapy initiation. Consequently > 80% of the patients received ganciclovir or valganciclovir therapy, often within the first 2 post-transplant weeks, with these medications by themselves suppressing the bone marrow and could have caused or contributed to the lymphopenia. Therefore, larger studies are required to confirm our observation and recently favored less cautious strategies for initiation for antiviral therapy^5, 33^ have to be considered by clinical programs. Fifth, some patients started preemptive therapy within one or two weeks post-transplant, so the low ALC on POD #7 could have been related to very early CMV infection or antiviral therapy. Despite these limitations, our findings were internally consistent and supported those of other similar studies.

## Conclusion

In conclusion, low ALC after HT was associated with a higher risk of early CMV infection. Close monitoring of ALC after HT may help to identify patients at a high risk of early CMV infection.

## Supplementary information

Supplementary Information.

## Data Availability

Study data are available from the corresponding author on reasonable request.
