# Supplementary material for: Post-transplant absolute lymphocyte count predicts early cytomegalovirus infection after heart transplantation
Source: Sci Rep. 2021 Jan 14;11:1426. doi: 10.1038/s41598-020-80790-4 (PMC7809401; doi:10.1038/s41598-020-80790-4)
Supplement: Supplementary file 1 — Supplementary Information. [file 41598_2020_80790_MOESM1_ESM.docx]

**Post-transplant absolute lymphocyte count predicts early cytomegalovirus infection after heart transplantation**

Minjae Yoon*, MD; Jaewon Oh*, MD, PhD; Kyeong-Hyeon Chun, MD; Chan Joo Lee, MD, PhD; Seok-Min Kang, MD, PhD

*Cardiology Division, Severance Cardiovascular Hospital, Cardiovascular Research Institute, Yonsei University College of Medicine, Seoul, South Korea*

* The first two authors contributed equally to this work.

**Corresponding author:** Seok-Min Kang, MD, PhD

**Supplementary Table S1.** Univariable and multivariable logistic regression analysis of risk factors for early CMV infection within 2 months of heart transplantation

|  | **Univariable analysis** | | | **Multivariable analysis**  **(Model 1)** | | | **Multivariable analysis**  **(Model 2)** | | |
| --- | --- | --- | --- | --- | --- | --- | --- | --- | --- |
|  | **OR** | **95% CI** | ***P*-value** | **OR** | **95% CI** | ***P*-value** | **OR** | **95% CI** | ***P*-value** |
| Age | 1.10 | 1.04–1.16 | 0.001 | 1.10 | 1.03–1.17 | 0.003 | 1.10 | 1.04–1.17 | 0.002 |
| Female | 0.78 | 0.22–2.70 | 0.692 | 2.39 | 0.45–12.85 | 0.310 | 2.08 | 0.41–10.50 | 0.375 |
| Decrease in pre-transplant ALC (per 100 cells/μL increment) | 1.04 | 0.94–1.14 | 0.476 |  |  |  |  |  |  |
| Decrease in ALC on POD #7  (per 100 cells/μL increment) | 1.12 | 0.98–1.27 | 0.097 |  |  |  |  |  |  |
| ALC < 500 on POD #7  versus ALC ≥500 cells/μL | 4.56 | 1.10–18.86 | 0.036 | 3.15 | 0.64–15.41 | 0.157 |  |  |  |
| ALC < 610 on POD #7  versus ALC ≥610 cells/μL | 3.20 | 0.89–11.48 | 0.074 |  |  |  |  | 0.44–8.34 | 0.393 |

ALC = absolute lymphocyte count, CI = confidence interval, CMV = cytomegalovirus, OR = oddds ratio, POD = postoperative day.

**Supplementary Table S2.** Univariable logistic regression analysis of total leukocyte count for early CMV infection within 1 month of heart transplantation

|  | **Univariable analysis** | | |
| --- | --- | --- | --- |
|  | **OR** | **95% CI** | ***P*-value** |
| Decrease in pre-transplant total leukocyte count (per 100 cells/μL increment) | 1.01 | 0.99–1.02 | 0.253 |
| Decrease in total leukocyte count on POD #7  (per 100 cells/μL increment) | 1.00 | 0.99–1.01. | 0.576 |
| Total leukocyte count < 4,000 cells/μL on pre-transplant | 1.63 | 0.44–5.98 | 0.462 |
| Total leukocyte count < 10,000 cells/μL on POD #7 | 0.98 | 0.32–0.296 | 0.969 |

CI = confidence interval, CMV = cytomegalovirus, OR = odds ratio, POD = postoperative day.

**FIGURES**

**
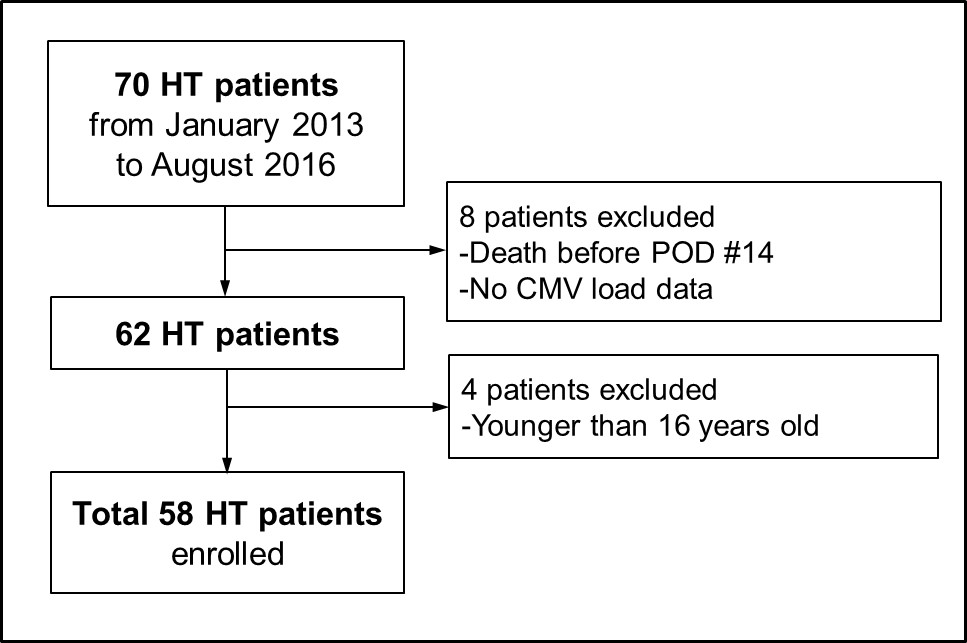
**

**Supplementary Figure S1. Patient enrollment in the study**

CMV = cytomegalovirus, HT = heart transplantation, POD = postoperative day.
